# A New Diagnostic Strategy for Polycystic Ovary Syndrome Combining Japanese and International Diagnostic Criteria Using Anti‐Müllerian Hormone

**DOI:** 10.1111/jog.70326

**Published:** 2026-05-06

**Authors:** Hiroki Noguchi, Takeshi Iwasa, Akira Iwase, Haruhiko Kanasaki, Fuminori Kimura, Koji Kugu, Kazuki Saito, Tsuyoshi Baba, Tetsuaki Hara, Toshiya Matsuzaki

**Affiliations:** ^1^ Department of Obstetrics and Gynecology, Graduate School of Biomedical Sciences Tokushima University Tokushima Japan; ^2^ Department of Obstetrics and Gynecology Gunma University Graduate School of Medicine Maebashi Japan; ^3^ Obstetrics and Gynecology, Faculty of Medicine Shimane University Matsue Japan; ^4^ Department of Obstetrics and Gynecology Nara Medical University Nara Japan; ^5^ Department of Pharmaceutical Sciences (Narita Campus) International University of Health and Welfare Chiba Japan; ^6^ Department of Perinatal and Maternal Medicine (Ibaraki), Graduate School Institute of Science Tokyo Tokyo Japan; ^7^ Department of Obstetrics and Gynecology Sapporo Medical University Sapporo Japan; ^8^ Division of Reproductive Medicine Hiroshima Prefectural Hospital Hiroshima Japan; ^9^ Department of Obstetrics and Gynecology Yoshinogawa Medical Center Tokushima Japan

**Keywords:** anti‐Müllerian hormone, antral follicle count, cut‐off value, diagnostic criteria, polycystic ovary syndrome

## Abstract

**Aim:**

We had defined the anti‐Müllerian hormone (AMH) cut‐off value level 2 aligned with the Rotterdam/International Evidence‐based Guideline (IEBG) for the Assessment and Management of polycystic ovary syndrome (PCOS) 2023 criteria. In this study, we evaluated the utility of AMH cut‐off value level 2 in patients who could not be diagnosed under the Japan Society of Obstetrics and Gynecology (JSOG) 2024 criteria due to the absence of endocrinological abnormalities and estimated the utility of a new diagnostic approach combining the JSOG 2024 and the Rotterdam/IEBG 2023 criteria.

**Methods:**

Through a nationwide survey in Japan, data were collected for 270 patients with irregular menstrual cycles and an antral follicle count of ≥ 10 to assess the new diagnostic approach.

**Results:**

Of 270 patients, 213 (78.9%) met the JSOG 2024 criteria due to the presence of endocrinological abnormalities. Of the remaining 57 patients (21.1%) who did not meet the JSOG 2024 criteria, 36 (63.2%) were additionally diagnosed with PCOS under the Rotterdam/IEBG 2023 criteria by applying elevated serum AMH (level 2). Consequently, the diagnostic rate of PCOS increased by 16.9% (obese/overweight: 9.8%, non‐obese/overweight: 19.7%), and the overall diagnostic rate reached 92.2%. The diagnostic rate of this new diagnostic approach was significantly higher than that of the JSOG 2024 or the Rotterdam/IEBG 2023 criteria alone. Patients additionally diagnosed by this new diagnostic approach were significantly older and had a higher prevalence of oligomenorrhea and lower prevalence of amenorrhea compared with those diagnosed under the JSOG 2024 criteria.

**Conclusion:**

Applying elevated serum AMH (level 2) based on the Rotterdam/IEBG 2023 criteria improved the diagnostic rate of PCOS, particularly in non‐obese/overweight patients and relatively older women with milder PCOS phenotypes. This new approach is practical, complementary, and can help overcome limitations of the diagnosis of PCOS and thus expand diagnostic opportunities.

## 1 Introduction

In 1935, Stein and Leventhal reported a series of disorders characterized by bilateral polycystic ovaries, oligo/amenorrhea, hirsutism, and obesity [1]. Subsequently, advances in hormonal assays and ultrasonographic techniques revealed that numerous patients exhibited milder clinical phenotypes with similar pathophysiological features [2]. Accordingly, this spectrum of disorders was collectively described as polycystic ovary syndrome (PCOS). PCOS is commonly associated with gynecological conditions such as infertility, endometrial hyperplasia, and endometrial cancer [3, 4]. Metabolic complications such as insulin resistance, type 2 diabetes, and metabolic syndrome [5, 6, 7]; dermatological symptoms such as hirsutism and acne [8]; and psychiatric disorders such as anxiety and depression are also associated with PCOS [9]. Therefore, it is essential to accurately identify women with PCOS in order to manage the condition effectively over the long term [10, 11].

Ovarian morphology has been considered an important component in the diagnosis of PCOS, and polycystic ovarian morphology (PCOM) has been consistently included in the Japanese diagnostic criteria since the first criteria were established in 1993 (Japan Society of Obstetrics and Gynecology [JSOG] 1993 criteria) [12, 13]. PCOM was also incorporated into the international diagnostic criteria in 2003 (Rotterdam 2003 criteria) and has remained a diagnostic component ever since [14, 15, 16]. Both the international and Japanese diagnostic criteria for PCOS were recently revised (i.e., Rotterdam/International Evidence‐based Guideline for the Assessment and Management of PCOS [IEBG] 2023 criteria [17] and JSOG 2024 criteria [18, 19, 20]). In both sets of diagnostic criteria, an elevated serum anti‐Müllerian hormone (AMH) level was adopted as a surrogate marker of PCOM.

The number of follicles in every growing stage is greater in women with PCOS than in healthy women, which is remarkable in the small antral follicle stage (2–8 mm in diameter) and the pre‐antral follicle stage (100 μm to 2 mm in diameter) [21, 22]. AMH is primarily secreted by granulosa cells in these stages, and the serum level reflects the stock of small antral and pre‐antral follicles [23, 24]. Therefore, serum AMH levels are two‐ to four‐fold higher in women with PCOS than in healthy women [25, 26, 27, 28, 29]. PCOM refers to the presence of many small follicles detected by transvaginal ultrasonography (TVUS). Specifically, it is evaluated by counting the number of small follicles measuring 2–9 mm in diameter, namely the antral follicle count (AFC). Therefore, the follicles counted in the evaluation of PCOM overlap with those that secrete AMH, although they do not completely coincide [27]. In our previous study, AFC showed a significant positive correlation with serum AMH levels and was identified as the factor with the greatest influence on serum AMH levels among the representative features of PCOS [26, 27]. The evaluation of PCOM for the diagnosis of PCOS can also be highly variable due to differences in training, specific methodology, and resolution of the TVUS equipment used [30]. In addition, discrimination of follicles measuring approximately 2 mm in diameter is difficult, and the measurements would be unreliable, particularly under the condition in which there is a large number of small follicles, such as in the case of PCOS. By contrast, the serum AMH level exhibits less variability and higher objectivity than AFC and remains relatively stable across the menstrual cycle [31, 32, 33]. Therefore, serum AMH level is considered a biochemical representation of ovarian findings in PCOS and is likely to play a very important role in the accurate diagnosis of PCOS.

According to the JSOG 2024 criteria, a diagnosis of PCOS requires the presence of all three of the following characteristics: (1) irregular menstrual cycle/chronic anovulation, (2) PCOM or elevated serum AMH level, and (3) hyperandrogenism or high luteinizing hormone (LH) level [18, 19, 20]. By contrast, according to the Rotterdam/IEBG 2023 criteria, a diagnosis of PCOS requires the presence of two or three of the following characteristics: (1) oligo‐ or anovulation, (2) clinical and/or biochemical signs of hyperandrogenism, and (3) PCOM or elevated serum AMH level [17]. Thus, the individual diagnostic characteristics are generally similar between the two sets of criteria, but the diagnostic requirements differ. In particular, in the JSOG 2024 criteria, each characteristic must exhibit high sensitivity for a positive diagnosis, whereas high specificity is required for a diagnosis according to the Rotterdam/IEBG 2023 criteria [34]. Accordingly, PCOM is defined in the JSOG 2024 criteria as the presence of ≥ 10 follicles measuring 2–9 mm in diameter in at least one ovary [18, 19, 20], whereas the Rotterdam/IEBG 2023 criteria define PCOM as the presence of ≥ 20 follicles and/or an increased ovarian volume (> 10 mL) [17]. In our previous study, AFC of ≥ 10 resulted in a sensitivity of ≥ 95% (AFC level 1), whereas an AFC of ≥ 20 resulted in a specificity of ≥ 95% (AFC level 2) [26, 27]. By contrast, no AMH cut‐off value is specified in the Rotterdam/IEBG 2023 criteria [17]. In our previous study, we established for the first time AMH cut‐off values for use in diagnostic criteria, and these values were applicable to the JSOG 2024 criteria with a sensitivity of ≥ 95% (AMH cut‐off value level 1) and the Rotterdam/IEBG 2023 criteria with a specificity of ≥ 95% (AMH cut‐off value level 2), based on nationwide survey data from Japan involving a large number of cases and taking into account patient age, body mass index (BMI), type of assay system, and structure of the diagnostic criteria [26, 27].

High LH is included only in the JSOG 2024 criteria to compensate for a relatively lower prevalence of hyperandrogenism in Japanese PCOS patients, among which obese/overweight patients (BMI ≥ 25 kg/m^2^) constitute as low as 26% of the total PCOS population [18, 19, 20, 35]. High LH is determined by both elevated basal LH level and elevated LH/follicle‐stimulating hormone (FSH) ratio. In obese/overweight PCOS patients, an elevated LH/FSH ratio alone is acceptable for diagnosis. High LH reflects the effects of chronic and non‐cyclical estrogen feedback on the central nervous system [36, 37]. Such disturbances in the complex feedback regulatory system of the hypothalamic–pituitary‐gonadal (HPG) axis are peculiar to PCOS and important as hyperandrogenism in the pathophysiology of PCOS. However, the detection rate for high LH is only 73.1% among Japanese PCOS patients [35]. In some cases, PCOS may be clinically suspected but cannot be definitively diagnosed under the JSOG 2024 criteria due to the absence of endocrinological abnormalities, such as hyperandrogenism and high LH. In such cases, applying elevated serum AMH (level 2) in order to diagnose PCOS according to the Rotterdam/IEBG 2023 criteria may provide a useful approach. Accordingly, we evaluated the efficacy of this new diagnostic strategy, that is, a combined use of the JSOG 2024 criteria and some of the Rotterdam/IEBG 2023 criteria in the diagnosis of PCOS using AMH cut‐off value level 2.

## 2 Methods

### 2.1 Research Design

This study was based on two studies previously reported in the *Journal of Obstetrics and Gynecology Research* [26, 35]. Although analyses in the present study were conducted using a subset of the dataset used in the previous studies, the research objectives of the present and previous studies differed. The first previous study focused on ovarian findings along with serum AMH levels and AFC [26], whereas the second previous study focused on endocrinological findings, including hyperandrogenism and high LH [35]. In the first previous study, AMH cut‐off value level 1 and AFC level 1 were established to align with the JSOG 2024 criteria, and AMH cut‐off value level 2 and AFC level 2 were established to align with the Rotterdam/IEBG 2023 criteria (Table 1) [26]. The present study, by contrast, focused on the diagnostic utility of combining the JSOG 2024 and the Rotterdam/IEBG 2023 criteria using AMH cut‐off value level 2 for the diagnosis of PCOS. Notice of the fact‐finding survey was sent via regular mail on September 15, 2022, and the survey began on September 16, 2022, with a response deadline of December 23, 2022. No incentives were provided for responding to the factual survey. Using the REDCap (Research Electronic Data Capture) data collection management system, the survey was implemented at 643 public and private university hospitals and assisted reproductive technology (ART) facilities registered with the JSOG. Data were summarized regarding age, BMI, irregular menstrual cycles, serum AMH level, PCOM, clinical hyperandrogenism, biochemical hyperandrogenism, and high LH, which was defined as both elevated basal LH level and elevated LH/FSH ratio in non‐obese/overweight patients, whereas elevated LH/FSH ratio alone was sufficient in obese/overweight patients. In Japan, an irregular menstrual cycle is defined as oligomenorrhea for cycles > 38 days but < 3 months, as secondary amenorrhea for the absence of menstruation for > 3 months, and as anovulatory cycle for normal cycle length without ovulation.

**TABLE 1:** AMH and AFC cut‐off values for the JSOG 2024 criteria (level 1) and the Rotterdam/IEBG 2023 criteria (level 2) established in our previous study.

		Level 1 (JSOG 2024 criteria)			Level 2 (Rotterdam/IEBG 2023 criteria)		
		20–29 years	30–34 years	35–39 years	20–29 years	30–34 years	35–39 years
Serum AMH level (ng/mL)	Access Lumipulse	4.4	3.1		9.9	7.3	6.0
	Elecsys	4.0	2.8		9.0	6.7	5.5
AFC		10			20		

*Note:* These cut‐off values were established in our previous study [26]. In the present study, the cut‐off value applicable to the JSOG 2024 criteria with a sensitivity of ≥ 95% was defined as level 1, whereas that applicable to the Rotterdam/IEBG 2023 criteria with a specificity of ≥ 95% was defined as level 2.

Abbreviations: AFC, antral follicle count; AMH, anti‐Müllerian hormone; IEBG, International Evidence‐based Guideline for the Assessment and Management of polycystic ovary syndrome; JSOG, Japan Society of Obstetrics and Gynecology.

The detection rate of hyperandrogenism, defined as the presence of either elevated serum T level or hirsutism, as well as the rates of high LH and elevated serum AMH (level 2) were calculated and compared. The diagnostic rate of PCOS using the JSOG 2024 criteria based on hyperandrogenism and/or high LH, and that using the Rotterdam/IEBG 2023 criteria based on hyperandrogenism and/or elevated serum AMH (level 2) were evaluated. In addition, the diagnostic rate of PCOS using the new diagnostic strategy combining the JSOG 2024 and the Rotterdam/IEBG 2023 criteria by applying elevated serum AMH (level 2) was evaluated. These three diagnostic rates were compared to evaluate the utility of the new diagnostic strategy, stratified by obese/overweight and non‐obese/overweight groups. Furthermore, the clinical characteristics of patients additionally diagnosed based on elevated serum AMH (level 2) according to the new diagnostic strategy were compared with those of patients diagnosed according to the JSOG 2024 criteria.

### 2.2 Subjects

The study population comprised 270 patients aged 20–39 years who had been diagnosed with PCOS at least once according to the JSOG 2007 criteria and/or the Rotterdam 2003 criteria between April 2020 and March 2022. The 10 most recent consecutive patients diagnosed or in whom treatment was initiated during the specified period were set as the maximum limit for each facility.

All 270 patients had irregular menstrual cycles and AFC of ≥ 10, but 57 of them had no endocrinological abnormalities. Detailed AFC data, rather than only whether AFC was ≥ 10, were available for only 122 patients.

### 2.3 Hormone Assays

Serum AMH levels were measured using a chemiluminescence immunoassay with Access AMH (Beckman Coulter Japan, K.K., Tokyo, Japan), an electrochemiluminescence immunoassay with the ECLusys AMH Plus assay (Roche Diagnostics, K.K., Tokyo, Japan), known as Elecsys AMH worldwide, and a chemiluminescent enzyme immunoassay with Lumipulse G AMH (Fujirebio Diagnostics Japan Inc., Tokyo, Japan). Among the three assay systems set up using the same serum samples, Lumipulse is the newest and only assay system that provides reliable correlation formulas. The correlation coefficients for the Lumipulse and other two assays were extremely high, with a value of 0.997 between the Access and Lumipulse systems and 0.988 between the Elecsys/ECLusys and Lumipulse systems, according to data generated during development of the Lumipulse assay. In the present study, serum AMH levels measured using the Access and Elecsys/ECLusys assays were converted to Lumipulse values using the following regression equations: Lumipulse = 1.0 × Access + 0.015 (ng/mL); Lumipulse = 1.1 × Elecsys/ECLusys −0.041 (ng/mL) [38].

Serum basal LH and FSH levels were primarily measured using Elecsys/ECLusys and the Architect chemiluminescence immunoassay (Abbott Japan Inc., Tokyo, Japan). As only a limited number of cases were analyzed using other assay systems, those cases were excluded from the analysis. For the Architect assay, the cut‐off values indicating high LH in the diagnosis of PCOS are LH level ≥ 7.1 mIU/mL and LH/FSH ratio ≥ 1.21, whereas cut‐offs for the Elecsys/ECLusys assay are LH level ≥ 9.9 mIU/mL and LH/FSH ratio ≥ 1.51 [18, 19, 20, 39]. LH and FSH levels measured using the Elecsys/ECLusys assay were converted to Architect assay values using the following regression equations: Architect LH = 0.778 × Elecsys/ECLusys LH −0.641 (mIU/mL); Architect FSH = 0.815 × Elecsys/ECLusys FSH + 0.161 (mIU/mL); and Architect LH/FSH ratio = 0.858 × Elecsys/ECLusys LH/FSH ratio −0.0436 (Passing‐Bablok regression analysis using samples from 106 patients with PCOS and 99 controls; LH: rs = 0.99, *p* < 0.01; FSH: rs = 0.94, *p* < 0.01; LH/FSH ratio: rs = 0.99, p < 0.01) [26, 35].

Serum T values were expressed in mass units, and assay‐related differences were minimal. Therefore, no conversion of serum T values between assay systems was performed in the present study. The cut‐off value for serum T was defined as the upper limit of the reference range for each assay system [18, 19, 20, 35].

### 2.4 Statistical Analysis

Continuous variables are presented as the mean ± standard deviation (SD), and categorical data are expressed as count (%). After considering variance and distribution, differences in continuous variables between two groups were analyzed using Student's *t*‐test, Welch's *t*‐test, or the Mann–Whitney *U* test. Differences between three groups were analyzed using the Kruskal–Wallis test. If significant differences were observed, post hoc multiple comparisons were performed using the Steel‐Dwass test. Differences in categorical data were compared using the chi‐squared test.

Significance was defined as *p* < 0.05. All statistical analyses were performed using Statcel4 (Statcel‐the Useful Addin Forms on Excel‐4th ed.; OMS Publishing, Tokorozawa, Japan).

### 2.5 Ethical Considerations

This study was approved by the Ethics Committee of Tokushima University Hospital on August 22, 2022 (approval number 4222) and conducted in accordance with the ethical standards instituted by the Committee. The survey used only anonymized data, and as data correspondence sheets were neither possessed nor received, it was not possible to identify any individual who participated in the study. In addition, detailed information regarding the survey was posted at each facility, and patients were provided an opportunity to refuse participation. This article does not include results for any study involving animals performed by any of the authors.

## 3 Results

Significant differences in clinical and endocrinological characteristics were observed between the obese/overweight and non‐obese/overweight groups (Table 2). In the non‐obese/overweight group, age was significantly lower, whereas basal LH level, basal FSH level, and LH/FSH ratio were significantly higher compared with the obese/overweight group. Serum total testosterone (T) level was numerically lower in the non‐obese/overweight group compared with that in the obese/overweight group (0.48 ng/mL vs. 0.52 ng/mL, respectively), but the difference was not statistically significant (*p* = 0.10). No significant differences between the two groups were observed in terms of serum AMH level or AFC.

**TABLE 2:** Comparison of clinical and endocrinological characteristics between the obese/overweight and non‐obese/overweight groups.

		Total (*n* = 270)	Obese/overweight (BMI ≥ 25 kg/m^2^) (*n* = 69)	Non‐obese/overweight (BMI < 25 kg/m^2^) (*n* = 201)	*p*
Age (years)		31.2 ± 3.8	32.2 ± 4.1	30.9 ± 3.6	< 0.01
BMI (kg/m^2^)		22.9 ± 4.8	29.5 ± 4.1	20.6 ± 2.1	< 0.01
	≥ 25	25.6% (69/270)	100% (69/69)	—	—
	≥ 30	8.5% (23/270)	33.3% (23/69)	—	—
Irregular menstrual cycle	Anovulatory cycle	27.0% (73/270)	30.4% (21/69)	25.9% (52/201)	0.46
	Oligomenorrhea	54.8% (148/270)	47.8% (33/69)	57.2% (115/201)	0.18
	Amenorrhea (WHO group II)^a^	18.1% (49/270)	21.7% (15/69)	16.9% (34/201)	0.37
Total testosterone (ng/mL)		0.49 ± 0.24	0.52 ± 0.24	0.48 ± 0.24	0.10
LH (Architect conversion) (mIU/mL)		10.0 ± 5.3	8.0 ± 4.2	10.7 ± 5.5	< 0.01
FSH (Architect conversion) (mIU/mL)		5.7 ± 1.7	5.3 ± 2.1	5.8 ± 1.5	< 0.01
LH/FSH (Architect conversion)		1.8 ± 0.9	1.6 ± 0.8	1.9 ± 0.9	< 0.05
AMH (Lumipulse conversion) (ng/mL)		12.0 ± 6.8	10.5 ± 5.2	12.5 ± 7.3	0.09
AFC^b^		29.2 ± 12.6	31.9 ± 17.5	28.2 ± 10.1	0.60
Mean ± SD					

*Note:* Data are presented as mean ± standard deviation or percentage (number/total).

Abbreviations: AFC, antral follicle count; AMH, anti‐Müllerian hormone; BMI, body mass index; FSH, follicle‐stimulating hormone; LH, luteinizing hormone.

^a^ Amenorrhea (WHO group II) means amenorrhea with evidence of estrogen production and normal levels of prolactin and FSH.

^b^ AFC data were available for only 122 patients in whom AFC was evaluated in detail rather than only whether it was ≥ 10.

The rate of elevated serum T level was 38.1%, and the prevalence of hirsutism was 11.5% (Table 3). The detection rate of hyperandrogenism, defined as the presence of either elevated serum T level or hirsutism, was 44.8%. In addition, the rates of high LH and elevated serum AMH (level 2) were 64.4% and 73.7%, respectively. The rate of high LH was significantly higher than that of hyperandrogenism (*p* < 0.01). Furthermore, the rate of elevated serum AMH (level 2) was significantly higher than those of hyperandrogenism (*p* < 0.01) and high LH (*p* < 0.05). Consequently, the diagnostic rate of PCOS was 78.9% using the JSOG 2024 criteria based on hyperandrogenism and/or high LH, whereas it was 82.2% using the Rotterdam/IEBG 2023 criteria based on hyperandrogenism and/or elevated serum AMH (level 2) (Table 3, Figure S1). There was no significant difference between these diagnostic rates. With regard to differences between subgroups, the prevalence of hirsutism was significantly lower (*p* < 0.01), and the detection rate of hyperandrogenism tended to be lower (*p* = 0.09), in the non‐obese/overweight group compared with the obese/overweight group (Table 3). In contrast, there were no significant differences between the two groups in the rates of high LH and elevated serum AMH (level 2). The diagnostic rate of PCOS using the JSOG 2024 criteria based on hyperandrogenism and/or high LH was significantly lower in the non‐obese/overweight group compared with the obese/overweight group (75.6% vs. 88.4%, *p* < 0.05) (Table 3, Figure S1). In contrast, there was no significant difference in the diagnostic rate of PCOS using the Rotterdam/IEBG 2023 criteria between the two groups.

**TABLE 3:** Detection rates of endocrinological abnormalities and diagnostic rates of polycystic ovary syndrome using the JSOG 2024 criteria, the Rotterdam/IEBG 2023 criteria, and the new diagnostic strategy combining these criteria.

		Total	Obese/overweight (BMI ≥ 25 kg/m^2^)	Non‐obese/overweight (BMI < 25 kg/m^2^)	*p*
Hyperandrogenism	(1) Elevated serum total testosterone level	38.1% (103/270)	42.0% (29/69)	36.8% (74/201)	0.44
	(2) Hirsutism	11.5% (31/270)	20.3% (14/69)	8.5% (17/201)	< 0.01
	(1) and/or (2)	44.8% (121/270)	53.6% (37/69)	41.8% (84/201)	0.09
High LH^a^		64.4% (174/270)^aa^	69.6% (48/69)^a^	62.7% (126/201)^aa^	0.30
Elevated serum AMH (level 2)^b^		73.7% (199/270)^bb, c^	78.3% (54/69)^bb^	72.1% (145/201)^bb, c^	0.32
Diagnostic rate of JSOG 2024 criteria	Hyperandrogenism and/or High LH	78.9% (213/270)	88.4% (61/69)	75.6% (152/201)	< 0.05
Diagnostic rate of Rotterdam/IEBG 2023 criteria	Hyperandrogenism and/or Elevated serum AMH (level 2)^b^	82.2% (222/270)	85.5% (59/69)	81.1% (163/201)	0.41
Diagnostic rate of the new diagnostic strategy combining JSOG 2024 and Rotterdam/IEBG 2023 criteria	Hyperandrogenism and/or High LH and/or Elevated serum AMH (level 2)^b^	92.2% (249/270)^dd, ee^	97.1% (67/69)^d, e^	90.5% (182/201)^dd, ee^	0.08

*Note:* The detection rate of high LH was significantly higher than that of hyperandrogenism (a: *p* < 0.05; aa: *p* < 0.01). The detection rate of elevated serum AMH (level 2) was significantly higher those that of hyperandrogenism (bb: *p* < 0.01) and high LH (c: *p* < 0.05). The diagnostic rate of polycystic ovary syndrome using the new diagnostic strategy combining JSOG 2024 and Rotterdam/IEBG 2023 was significantly higher than that using JSOG 2024 (d: *p* < 0.05; dd: *p* < 0.01) and Rotterdam/IEBG 2023 (e: *p* < 0.05; ee: *p* < 0.01).

Abbreviations: AMH, anti‐Müllerian hormone; BMI, body mass index; FSH, follicle‐stimulating hormone; IEBG, International Evidence‐based Guideline for the Assessment and Management of polycystic ovary syndrome; JSOG, Japan Society of Obstetrics and Gynecology; LH, luteinizing hormone.

^a^ High LH was determined based on both an elevated basal LH level and elevated LH/FSH ratio, exceeding a mean value +1 SD of normal females, respectively. In obese/overweight cases (BMI ≥ 25 kg/m^2^), an elevated LH/FSH ratio alone is acceptable for diagnosis.

^b^ Elevated serum AMH (level 2) was defined as an AMH level above the cut‐off value level 2 with a specificity of ≥ 95% for the Rotterdam/IEBG 2023 criteria (Table 1) [26].

**FIGURE 1:**
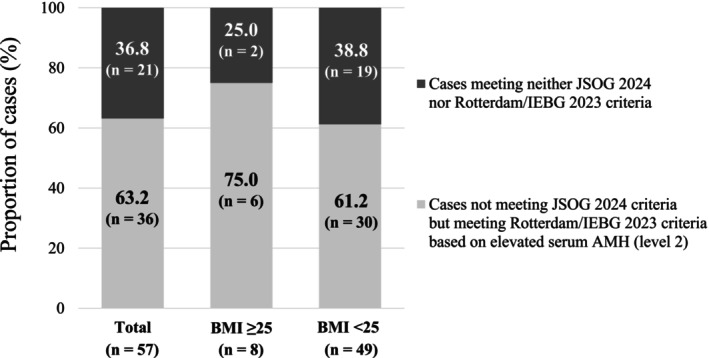
Among 57 patients who did not meet JSOG 2024 criteria due to the absence of endocrinological abnormalities, 36 patients (63.2%) were additionally diagnosed with PCOS according to the Rotterdam/IEBG 2023 criteria applying elevated serum AMH (level 2), defined as an AMH level above the cut‐off value level 2 with a specificity of ≥ 95% (Table 1) [26] (75.0% and 61.2% in the obese/overweight and non‐obese/overweight groups, respectively).

**FIGURE 2:**
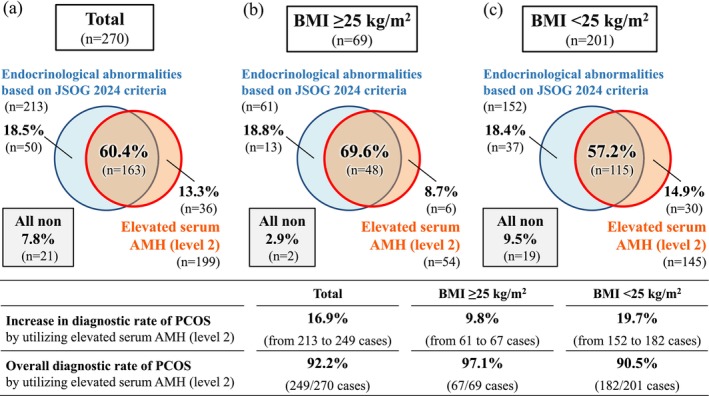
Among 270 patients who had been diagnosed at least once with PCOS according to the JSOG 2007 criteria and/or the Rotterdam 2003 criteria, 213 patients (78.9%) had endocrinological abnormalities and met the JSOG 2024 criteria. Of the remaining 57 patients who did not meet the JSOG 2024 criteria, 36 patients were additionally diagnosed with PCOS according to the Rotterdam/IEBG 2023 criteria by applying elevated serum AMH (level 2), defined as an AMH level above the cut‐off value level 2 with a specificity of ≥ 95% (Table 1) [26]. Consequently, the diagnostic rate of PCOS increased by 16.9% (obese/overweight: 9.8%; non‐obese/overweight: 19.7%), and the overall diagnostic rate reached 92.2% (obese/overweight: 97.1%; non‐obese/overweight: 90.5%).

Among 270 patients who had been diagnosed at least once with PCOS according to the JSOG 2007 criteria, 213 patients (78.9%) had endocrinological abnormalities (hyperandrogenism and/or high LH) and met the JSOG 2024 criteria, as described above (Table 3). Namely, 57 patients (21.1%) of the 270 patients did not meet the JSOG 2024 criteria due to the absence of endocrinological abnormalities. Of these 57 patients, 36 patients (63.2%) were additionally diagnosed with PCOS according to the Rotterdam/IEBG 2023 criteria applying elevated serum AMH (level 2) (Figure 1). After including these 36 patients, the number of patients diagnosed with PCOS increased from 213 to 249. This indicated that the diagnostic rate of PCOS increased by 16.9% and that the overall diagnostic rate of the new diagnostic strategy combining the JSOG 2024 and the Rotterdam/IEBG 2023 criteria reached 92.2% (249/270 patients) (Figure 2). The diagnostic rate of PCOS increased by 9.8% (from 61 to 67 patients) in the obese/overweight group and by 19.7% (from 152 to 182 patients) in the non‐obese/overweight group. Consequently, the overall diagnostic rate of the new diagnostic strategy combining the JSOG 2024 and the Rotterdam/IEBG 2023 criteria exceeded 90% in both groups (obese/overweight: 97.1%; non‐obese/overweight: 90.5%). No significant difference in the diagnostic rate was observed between the two groups. The diagnostic rate of the new diagnostic strategy was significantly higher than that of the JSOG 2024 criteria (overall, non‐obese/overweight, *p* < 0.01; obese/overweight, *p* < 0.05) and that of the Rotterdam/IEBG 2023 criteria (overall, non‐obese/overweight, *p* < 0.01; obese/overweight, *p* < 0.05) (Table 3).

Compared with patients diagnosed according to the JSOG 2024 criteria, patients additionally diagnosed based on elevated serum AMH (level 2) had the following characteristics: 1. Older (*p* < 0.01) (Table 4), 2. Higher prevalence of oligomenorrhea (*p* < 0.01) and lower prevalence of amenorrhea (*p* < 0.01) (Table 4), 3. Lower basal LH level (*p* < 0.01) and lower LH/FSH ratio (p < 0.01) (Table S1), and 4. With regard to the timing of blood sampling for measurement of basal LH and FSH levels, days 2–3 of the menstrual cycle was more frequent (*p* < 0.01), whereas the initial consultation involving a chief complaint of irregular menstrual cycle was less frequent (*p* < 0.05) (Table S1).

**TABLE 4:** Comparison of clinical characteristics between cases meeting the JSOG 2024 criteria and cases additionally diagnosed according to the Rotterdam/IEBG 2023 criteria by applying elevated serum AMH (level 2).

		Cases meeting JSOG 2024 criteria	Cases not meeting JSOG 2024 criteria but meeting Rotterdam/IEBG 2023 criteria based on elevated serum AMH (level 2)^a^	*p*
Number of cases		213	36	
Age (mean ± SD) (years)		30.9 ± 3.8	33.3 ± 2.8	< 0.01
BMI (mean ± SD) (kg/m^2^)		23.1 ± 4.9	22.6 ± 4.7	0.69
	≥ 25	28.6% (61/213)	16.7% (6/36)	0.13
	≥ 30	8.9% (19/213)	8.3% (3/36)	0.91
Irregular menstrual cycles	Anovulatory cycle	27.7% (59/213)	22.2% (8/36)	0.49
	Oligomenorrhea	49.8% (106/213)	75.0% (27/36)	< 0.01
	Amenorrhea (WHO group II)^b^	22.5% (48/213)	2.8% (1/36)	< 0.01

Abbreviations: AMH, anti‐Müllerian hormone; BMI, body mass index; IEBG, International Evidence‐based Guideline for the Assessment and Management of polycystic ovary syndrome; JSOG, Japan Society of Obstetrics and Gynecology; SD, standard deviation.

^a^ Elevated serum AMH (level 2) was defined as an AMH level above the cut‐off value level 2 with a specificity of ≥ 95% for the Rotterdam/IEBG 2023 criteria (Table 1) [26].

^b^ Amenorrhea (WHO group II) was defined as amenorrhea with evidence of estrogen production and normal levels of prolactin and FSH.

Among the 122 patients in which AFC was determined in addition to measurement of serum AMH, 30 patients (24.6%) showed no endocrinological abnormalities according to the JSOG 2024 criteria (Figure S2). Of these 30 patients, 21 patients (70.0%) were diagnosed with PCOS according to the Rotterdam/IEBG 2023 criteria applying elevated serum AMH (level 2) (Figure S2a). Similarly, of the 30 patients without endocrinological abnormalities based on the JSOG 2024 criteria, 21 patients (70.0%) were diagnosed with PCOS according to the Rotterdam/IEBG 2023 criteria applying AFC (level 2) (Figure S2b).

## 4 Discussion

Cases have been reported in Japan in which PCOS is clinically suspected but cannot be definitively diagnosed under the JSOG 2024 criteria due to the absence of endocrinological abnormalities. We found that applying elevated serum AMH (level 2) in order to diagnose PCOS according to the Rotterdam/IEBG 2023 criteria increased the diagnostic rate of PCOS in such cases by 16.9%, resulting in an overall diagnostic rate of 92.2%. The diagnostic rate of this new diagnostic approach—combining the Rotterdam/IEBG 2023 criteria using AMH level [level 2] with JSOG 2024 criteria—was significantly higher than that of the JSOG 2024 or the Rotterdam/IEBG 2023 criteria alone. These findings suggest that this new approach is a useful strategy for diagnosing PCOS in Japanese women.

The JSOG 2024 criteria require the presence of all three diagnostic characteristics for a diagnosis of PCOS [18, 19, 20]. In Japanese women with PCOS, the prevalences of overweight/obesity and hyperandrogenism, including hirsutism, are relatively low compared with women in other countries [35, 40, 41, 42, 43, 44, 45]. Moreover, even among obese or overweight Japanese women with PCOS, the prevalence of hirsutism is lower than that reported in the literature for Caucasian women with PCOS, despite comparable BMI values [46]. Furthermore, because basal LH levels are affected by the timing of measurement and pulsatile secretion patterns [35, 47, 48], high LH may not always be detected. Consequently, a subset of patients exists in whom PCOS is clinically suspected but cannot be definitively diagnosed under the JSOG 2024 criteria due to the absence of endocrinological abnormalities, such as hyperandrogenism and high LH. In the present study, 21.1% of all PCOS patients could not be diagnosed as PCOS according to the JSOG 2024 criteria. In contrast to serum LH or T levels, serum AMH levels are not affected by BMI or the timing of measurement and did not exhibit a relationship with serum LH or T levels [26, 27, 33]. Therefore, serum AMH might be a suitable criterion even in patients without endocrinological abnormalities. In the present study, the rate of elevated serum AMH (level 2) was significantly higher than those of high LH and hyperandrogenism (elevated serum T level and/or hirsutism). Furthermore, 63.2% of patients who had no endocrinological abnormalities according to the JSOG 2024 criteria were diagnosed with PCOS according to the Rotterdam/IEBG 2023 criteria using elevated serum AMH (level 2). Therefore, it would be beneficial to determine serum levels of LH, FSH, T and AMH at once on the day of the first visit for patients having chronic irregular menstrual cycles, in order to diagnose PCOS immediately and with a high degree of certainty.

Patients additionally diagnosed based on elevated serum AMH (level 2) had a significantly higher prevalence of oligomenorrhea and lower prevalence of amenorrhea compared with those diagnosed according to the JSOG 2024 criteria. These patients also had lower serum T and LH levels, suggesting milder disturbances in the feedback regulatory system of the HPG axis and less severe endocrinological abnormalities. Furthermore, these patients were significantly older, suggesting that endocrinological abnormalities may improve with age. Longitudinal follow‐up of women with PCOS has shown gradual declines in serum T, LH, and AMH levels, which reflects age‐related depletion of primordial ovarian follicles and follicular recruitment [28, 49], leading to attenuation of chronic and non‐cyclic estrogen feedback within the HPG axis. Therefore, the diagnosis of relatively older patients with milder PCOS phenotypes requires age‐stratified cut‐off values for each hormone. In the JSOG 2024 criteria, age‐stratified cut‐off values are defined for AMH, whereas uniform cut‐off values are applied to serum T and LH levels and LH/FSH ratio, regardless of age [18, 19, 20, 26, 27, 39]. Accordingly, additional use of the AMH cut‐off value level 2 would be useful for diagnosing relatively older patients with milder PCOS phenotypes. With regard to the timing of blood sampling for measurement of basal LH and FSH levels, there were some differences between the patient groups. Days 2–3 of the menstrual cycle was more frequent, whereas the initial consultation (involving a chief complaint of irregular menstrual cycle after confirming the absence of follicles measuring ≥ 1 cm in diameter) was less frequent in patients additionally diagnosed based on elevated serum AMH (level 2) than in patients diagnosed according to the JSOG 2024 criteria. As described above, basal LH levels are affected by the timing of measurement because serum LH levels vary according to steroidal feedback [35]. In our previous study, the rates of elevated basal LH level and LH/FSH ratio after day 10 of the menstrual cycle were higher than on days 1–10 of the menstrual cycle [47]. Similarly, in the data from the nationwide survey conducted in 2022, the rates of elevated basal LH level and LH/FSH ratio were higher at the initial consultation than on days 2–3 or 4–6 of the menstrual cycle [35]. In the present study, high LH might not be detected in some of the patients who did not meet the JSOG 2024 criteria due to an inappropriate timing of blood sampling. Therefore, it might be appropriate for the efficient assessment of high LH to measure the serum LH level at the initial consultation or after day 10 of the cycle, and retesting would also be advisable when high LH is not detected.

With regard to differences associated with body stature in the present study, the prevalence of hirsutism was significantly lower in non‐obese/overweight patients than in obese/overweight patients, consistent with previous reports [35]. In obese/overweight patients with insulin resistance, compensatory hyperinsulinemia enhances androgen production in ovarian theca cells [37, 50, 51], which may partially explain the higher prevalence of hirsutism in this group. Although previous studies have reported significantly lower serum T levels in non‐obese/overweight patients than in obese/overweight patients [35], this difference did not reach statistical significance in the present study, possibly due to differences in sample size or study population. Consequently, although the difference in the rate of high LH level between the two groups did not reach statistical significance, the detection rate of endocrinological abnormalities based on the JSOG 2024 criteria, including elevated serum T level, hirsutism, and high LH level, was significantly lower in the non‐obese/overweight group than in the obese/overweight group. In the non‐obese/overweight group, the detection rate of endocrinological abnormalities was relatively low, at 75.6%, suggesting that nearly one‐fourth of cases in which PCOS is clinically suspected cannot be definitively diagnosed according to JSOG 2024 criteria. This represents a clinically significant concern. By contrast, no significant difference in serum AMH levels was observed between obese/overweight and non‐obese/overweight patients in the present study, consistent with previous reports [26]. The increase in the diagnostic rate of PCOS using serum AMH level 2 was numerically greater in the non‐obese/overweight group than in the obese/overweight group, although this difference did not reach statistical significance (19.7% vs. 9.8%, *p* = 0.13). Moreover, in our previous single‐center study, this increase was significantly greater in the non‐obese/overweight group than in the obese/overweight group (16.4% vs. 2.4%, *p* < 0.05) [29]. Consequently, even in non‐obese/overweight patients, the overall diagnostic rate of PCOS achieved using this new approach in the present study exceeded 90%, despite a low detection rate of endocrinological abnormalities in this population. Furthermore, although the diagnostic rate of the JSOG 2024 criteria was significantly lower in the non‐obese/overweight group than in the obese/overweight group, the diagnostic rate of this new approach did not differ significantly between these groups. Therefore, in non‐obese/overweight patients, particularly North East Asian women, including Japanese women, the application of elevated serum AMH (level 2) to diagnose PCOS according to the Rotterdam/IEBG 2023 criteria represents a useful diagnostic approach. If this new approach is incorporated into the JSOG criteria, it may be formulated as follows: the presence of all three of the following is required for a diagnosis: (1) irregular menstrual cycle/chronic anovulation, (2) PCOM or elevated serum AMH (level 1), and (3) hyperandrogenism or high LH or elevated serum AMH (level 2).

Among cases in which both the serum AMH level and AFC were determined, cases involving patients diagnosed with PCOS according to the Rotterdam/IEBG 2023 criteria applying elevated serum AMH (level 2) did not completely overlap with cases of patients diagnosed applying PCOM with AFC ≥ 20 (AFC level 2), but the same number of patients were diagnosed using the two approaches. AFC is determined based on the number of follicles measuring 2–9 mm in diameter. By contrast, AMH is primarily secreted by granulosa cells of preantral and small antral follicles (100 μm to 8 mm in diameter) in the ovary [24]. Therefore, although not completely concordant, the follicles evaluated in determining the AFC partially overlap with those that secrete AMH [27]. In our previous study using multivariate regression analysis, serum AMH levels were most strongly affected by AFC [26]. Accordingly, AMH has been adopted as a surrogate marker of AFC for PCOM in both the Rotterdam/IEBG 2023 and the JSOG 2024 criteria [12, 13, 14, 15]. The cut‐off values for serum AMH (level 2) and AFC (level 2) used in the present study were established in our previous study with a specificity of ≥ 95% to align with the Rotterdam/IEBG 2023 criteria [26, 27]. Accordingly, it is reasonable that the same number of patients were diagnosed with PCOS in the present study according to the Rotterdam/IEBG 2023 criteria when applying serum AMH (level 2) and AFC (level 2). The results of PCOM evaluation in the diagnosis of PCOS can also vary considerably due to differences in operator training, specific methodologies, and resolution of TVUS equipment [30, 52, 53]. In our previous nationwide survey, AFC exhibited significantly greater inter‐institutional variability than AMH level [26]. Because it is difficult to discriminate follicles with a diameter of approximately 2 mm, assessments of AFC would be unreliable, especially under conditions characterized by a large number of small follicles, such as occurs with PCOS. In particular, accurate determination of an AFC of ≥ 20 seems difficult to achieve and varies by practitioner. Therefore, a finding of elevated serum AMH (level 2) may represent a more practical criterion than determination of AFC (level 2) in routine clinical practice. Because serum AMH measurement does not require TVUS equipment, PCOS can be diagnosed in clinical departments other than obstetrics and gynecology [27]. In addition, measuring the AMH level is useful in patients without a history of sexual intercourse. Accordingly, serum AMH measurement should increase the likelihood of diagnosing PCOS.

This study has several limitations. As the study involved a nationwide survey in Japan, it is necessary to take into account the impact of racial and ethnic differences. In addition, the nationwide survey data used in this study did not include variables related to metabolic features. Therefore, the risk of various health problems including metabolic complications remains a concern for future studies. AFC was not assessed using identical equipment or by a single investigator. Because this was a nationwide survey, the evaluation of PCOM based on AFC for the diagnosis of PCOS may have been affected by inter‐institutional and inter‐observer variability. Furthermore, the modified Ferriman‐Gallwey score might not be consistently used for the assessment of hirsutism because this survey was conducted before hirsutism was incorporated into the JSOG criteria. Accordingly, the assessment of hirsutism was based on the judgment of each attending physician. Finally, not all parameters were evaluated for every case and facility, resulting in incomplete data for some cases.

In conclusion, combined use of the JSOG 2024 criteria and some of the Rotterdam/IEBG 2023 criteria applying elevated serum AMH (level 2) substantially improved the diagnostic rate of PCOS compared with the JSOG 2024 or the Rotterdam/IEBG 2023 criteria alone. This approach was particularly effective in non‐obese/overweight patients and relatively older women with milder PCOS phenotypes, in whom conventional endocrinological markers are less frequently detected. The new diagnostic strategy represents a practical and complementary diagnostic approach for PCOS and may help overcome some of the limitations in diagnosing PCOS while expanding the likelihood of diagnosis.

## Author Contributions

**Hiroki Noguchi:** conceptualization; methodology; data curation; formal analysis; visualization; writing – original draft. **Takeshi Iwasa:** conceptualization; methodology; project administration; supervision; writing – review and editing. **Akira Iwase:** project administration; supervision; writing – review and editing. **Haruhiko Kanasaki:** investigation; data curation; writing – review and editing. **Fuminori Kimura:** investigation; data curation; writing – review and editing. **Koji Kugu:** supervision; writing – review and editing. **Kazuki Saito:** investigation; data curation; writing – review and editing. **Tsuyoshi Baba:** investigation; data curation; writing – review and editing. **Tetsuaki Hara:** supervision; writing – review and editing. **Toshiya Matsuzaki:** conceptualization; methodology; project administration; supervision; writing – review and editing.

## Funding

The authors have nothing to report.

## Disclosure

The authors have nothing to report.

## Ethics Statement

All the procedures were followed in accordance with the ethical standards of the responsible committees on human experimentation (institutional and national) and with the Helsinki Declaration of 1964 and its later amendments. This study was approved by the Ethics Committee of Tokushima University Hospital on August 22, 2022 (Approval Number 4222). This survey used only anonymized data, and we posted detailed information about the survey at each facility and provided an opportunity for patients to refuse. This article does not contain any study with animal participants that have been performed by any of the authors.

## Consent

Written informed consent was not obtained from individual participants because this study used anonymized data collected through a nationwide survey and was conducted using an opt‐out approach approved by the Ethics Committee. Detailed study information was disclosed at each participating facility, and participants were given the opportunity to refuse participation. No patient‐identifiable information is included in this article.

## Conflicts of Interest

Dr. Iwasa Takeshi, Dr. Kanasaki Haruhiko, Dr. Kimura Fuminori, and Dr. Kugu Koji are Editorial Board members of JOGR Journal and co‐authors of this article. To minimize bias, they were excluded from all editorial decision making related to the acceptance of this article for publication.

## Supporting information


**Figure S1:** Proportions of patients diagnosed with PCOS according to the JSOG 2024 criteria and the Rotterdam/IEBG 2023 criteria in the overall population, the obese/overweight group (BMI ≥ 25 kg/m^2^), and the non‐obese/overweight group (BMI < 25 kg/m^2^). According to the JSOG 2024 criteria, 78.9% of the overall population was diagnosed with PCOS (88.4% and 75.6% in the obese/overweight and non‐obese/overweight groups, respectively). 13.3% was additionally diagnosed with PCOS according to the Rotterdam/IEBG 2023 criteria applying elevated serum AMH (level 2), defined as an AMH level above the cut‐off value level 2 with a specificity of ≥ 95% (Table 1) [26] (8.7% and 14.9% in the obese/overweight and non‐obese/overweight groups, respectively) (a). According to the Rotterdam/IEBG 2023 criteria based on elevated serum AMH (level 2), 73.7% of the overall population was diagnosed with PCOS (78.3% and 72.1% in the obese/overweight and non‐obese/overweight groups, respectively). 18.5% was additionally diagnosed with PCOS according to the JSOG 2024 criteria based on hyperandrogenism and/or high LH (18.8% and 18.4% in the obese/overweight and non‐obese/overweight groups, respectively) (b). According to the Rotterdam/IEBG 2023 criteria based on elevated serum AMH (level 2) and/or hyperandrogenism, 82.2% of the overall population was diagnosed with PCOS (85.5% and 81.1% in the obese/overweight and non‐obese/overweight groups, respectively). 10.0% was additionally diagnosed with PCOS according to the JSOG 2024 criteria based on high LH (11.6% and 9.5% in the obese/overweight and non‐obese/overweight groups, respectively) (c).

**Figure S2:** Among the 122 patients in whom, in addition to serum AMH levels, AFC was evaluated in detail rather than only whether it was ≥ 10, 30 patients showed no endocrinological abnormalities according to the JSOG 2024 criteria. Among these patients, 21 patients were diagnosed with PCOS according to the Rotterdam/IEBG 2023 criteria applying elevated serum AMH (level 2), defined as an AMH level above the cut‐off value level 2 with a specificity of ≥ 95% (Table 1) [26] (a). Similarly, among the 30 patients without endocrinological abnormalities based on the JSOG 2024 criteria, 21 patients were diagnosed with PCOS according to the Rotterdam/IEBG 2023 criteria applying AFC (level 2), defined as an AFC of ≥ 20 with a specificity of ≥ 95% (b). The same number of patients were diagnosed with PCOS according to the Rotterdam/IEBG 2023 criteria when applying serum AMH (level 2) and AFC (level 2) (c).

**Table S1:** Comparison of endocrine characteristics among cases meeting the JSOG 2024 criteria, cases additionally diagnosed according to the Rotterdam/IEBG 2023 criteria by applying elevated serum AMH (level 2), and cases meeting neither the JSOG 2024 nor the Rotterdam/IEBG 2023 criteria.

## Data Availability

This study was approved by the Ethics Committee of Tokushima University Hospital on August 22, 2022 (approval number 4222) and conducted in accordance with the ethical standards of the Committee. The dataset analyzed in this study contains clinical data collected through a nationwide multicenter survey. Public deposition of these data is not permitted because the study protocol and participant information process did not include consent for public data sharing, and data sharing through a public repository was not explained in advance at each participating institution. Even though the data were anonymized, public release of the dataset may compromise participant privacy and may not comply with the ethical approval conditions for this study. Therefore, the data are not publicly available in a repository. The data from this study may be considered for sharing by the corresponding author upon reasonable request only if such sharing is consistent with ethical and legal requirements and has been approved by the relevant ethics committees and participating institutions.
