# Supplementary material for: A New Diagnostic Strategy for Polycystic Ovary Syndrome Combining Japanese and International Diagnostic Criteria Using Anti‐Müllerian Hormone
Source: J Obstet Gynaecol Res. 2026 May 6;52:e70326. doi: 10.1111/jog.70326 (PMC13149773; doi:10.1111/jog.70326)
Supplement: Supplementary file 1 — Figure S1: Proportions of patients diagnosed with PCOS according to the JSOG 2024 criteria and the Rotterdam/IEBG 2023 criteria in the overall population, the obese/overweight group (BMI ≥ 25 kg/m2), and the non‐obese/overweight group (BMI < 25 kg/m2). According to the JSOG 2024 criteria, 78.9% of the overall population was diagnosed with PCOS (88.4% and 75.6% in the obese/overweight and non‐obese/overweight groups, respectively). 13.3% was additionally diagnosed with PCOS according to the Rotterdam/IEBG 2023 criteria applying elevated serum AMH (level 2), defined as an AMH level above the cut‐off value level 2 with a specificity of ≥ 95% (Table 1) [26] (8.7% and 14.9% in the obese/overweight and non‐obese/overweight groups, respectively) (a). According to the Rotterdam/IEBG 2023 criteria based on elevated serum AMH (level 2), 73.7% of the overall population was diagnosed with PCOS (78.3% and 72.1% in the obese/overweight and non‐obese/overweight groups, respectively). 18.5% was additionally diagnosed with PCOS according to the JSOG 2024 criteria based on hyperandrogenism and/or high LH (18.8% and 18.4% in the obese/overweight and non‐obese/overweight groups, respectively) (b). According to the Rotterdam/IEBG 2023 criteria based on elevated serum AMH (level 2) and/or hyperandrogenism, 82.2% of the overall population was diagnosed with PCOS (85.5% and 81.1% in the obese/overweight and non‐obese/overweight groups, respectively). 10.0% was additionally diagnosed with PCOS according to the JSOG 2024 criteria based on high LH (11.6% and 9.5% in the obese/overweight and non‐obese/overweight groups, respectively) (c). [file JOG-52-0-s001.pptx]

## Slide 1
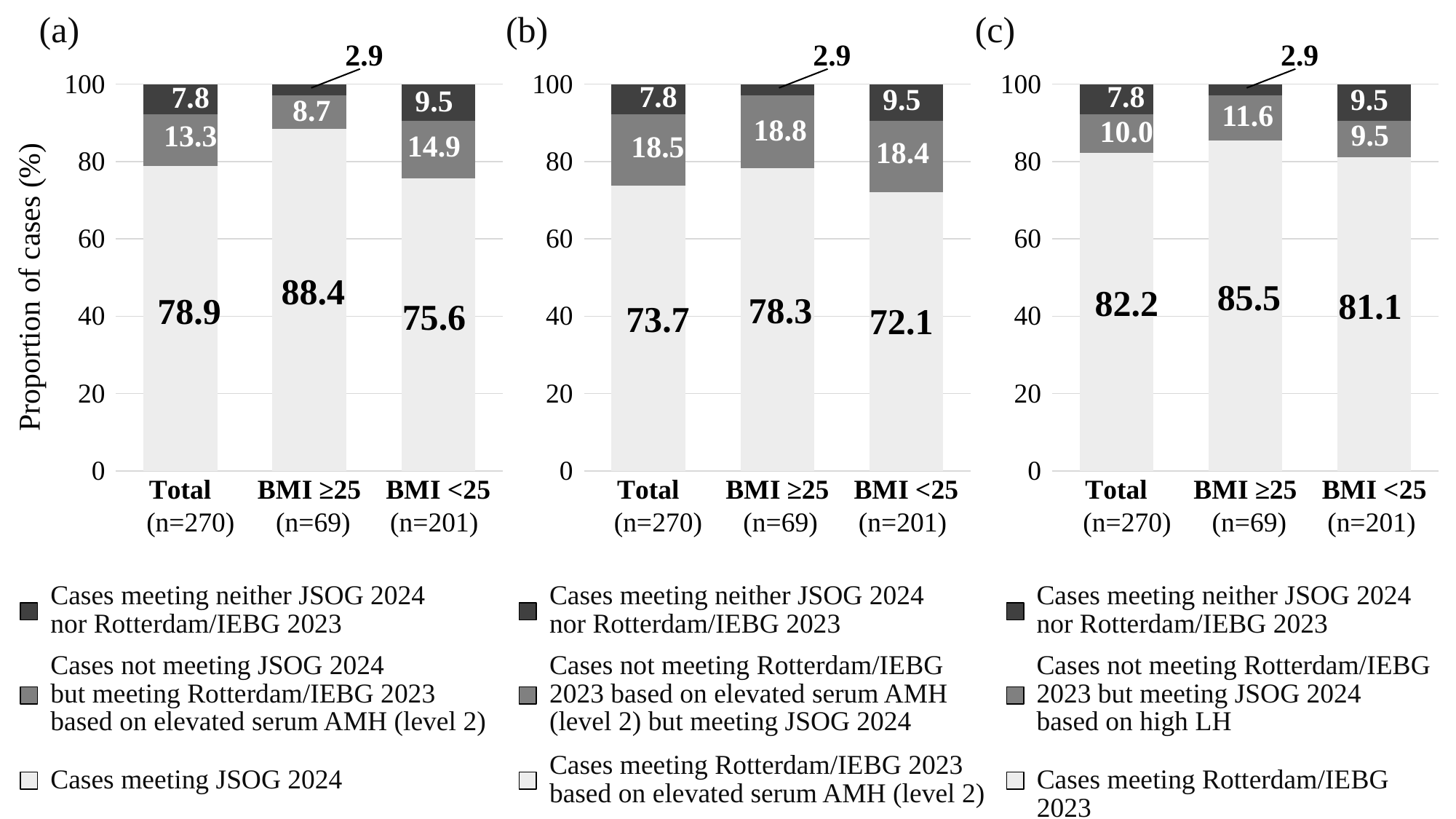

(a)
(b)
(c)
2.9
2.9
2.9
### Chart
| Category | JSOG2024 PCOS | Rotterdam/IEBG2023 PCOS
based on AMH,
but not meeting JSOG2024 | Cases not meeting JSOG2024,
nor Rotterdam/IEBG2023
based on AMH |
|---|---|---|---|
| Total | 78.9 | 13.3 | 7.8 |
| BMI ≥25 | 88.39999999999999 | 8.7 | 2.9 |
| BMI <25 | 75.6 | 14.9 | 9.5 |
### Chart
| Category | JSOG2024 PCOS | Rotterdam/IEBG2023 PCOS
based on AMH,
but not meeting JSOG2024 | Cases not meeting JSOG2024,
nor Rotterdam/IEBG2023
based on AMH |
|---|---|---|---|
| Total | 73.70370370370371 | 18.51851851851852 | 7.777777777777778 |
| BMI ≥25 | 78.26086956521739 | 18.84057971014493 | 2.898550724637681 |
| BMI <25 | 72.13930348258707 | 18.407960199004975 | 9.45273631840796 |
### Chart
| Category | JSOG2024 PCOS | Rotterdam/IEBG2023 PCOS
based on AMH,
but not meeting JSOG2024 | Cases not meeting JSOG2024,
nor Rotterdam/IEBG2023
based on AMH |
|---|---|---|---|
| Total | 82.22222222222221 | 10.0 | 7.777777777777778 |
| BMI ≥25 | 85.5072463768116 | 11.594202898550725 | 2.898550724637681 |
| BMI <25 | 81.09452736318407 | 9.45273631840796 | 9.45273631840796 |7.8
7.8
7.8
9.5
9.5
9.5
8.7
11.6
18.8
10.0
9.5
13.3
14.9
18.5
18.4
88.4
Proportion of cases (%)
85.5
82.2
81.1
78.3
78.9
75.6
73.7
72.1
(n=270)
(n=69)
(n=201)
(n=270)
(n=69)
(n=201)
(n=270)
(n=69)
(n=201)
Cases meeting neither JSOG 2024
nor Rotterdam/IEBG 2023
Cases meeting neither JSOG 2024
nor Rotterdam/IEBG 2023
Cases meeting neither JSOG 2024
nor Rotterdam/IEBG 2023
Cases not meeting JSOG 2024
but meeting Rotterdam/IEBG 2023
based on elevated serum AMH (level 2)
Cases not meeting Rotterdam/IEBG
2023 based on elevated serum AMH (level 2) but meeting JSOG 2024
Cases not meeting Rotterdam/IEBG
2023 but meeting JSOG 2024
based on high LH
Cases meeting Rotterdam/IEBG 2023
based on elevated serum AMH (level 2)
Cases meeting JSOG 2024
Cases meeting Rotterdam/IEBG 2023
