# Supplementary material for: A New Diagnostic Strategy for Polycystic Ovary Syndrome Combining Japanese and International Diagnostic Criteria Using Anti‐Müllerian Hormone
Source: J Obstet Gynaecol Res. 2026 May 6;52:e70326. doi: 10.1111/jog.70326 (PMC13149773; doi:10.1111/jog.70326)
Supplement: Supplementary file 2 — Figure S2: Among the 122 patients in whom, in addition to serum AMH levels, AFC was evaluated in detail rather than only whether it was ≥ 10, 30 patients showed no endocrinological abnormalities according to the JSOG 2024 criteria. Among these patients, 21 patients were diagnosed with PCOS according to the Rotterdam/IEBG 2023 criteria applying elevated serum AMH (level 2), defined as an AMH level above the cut‐off value level 2 with a specificity of ≥ 95% (Table 1) [26] (a). Similarly, among the 30 patients without endocrinological abnormalities based on the JSOG 2024 criteria, 21 patients were diagnosed with PCOS according to the Rotterdam/IEBG 2023 criteria applying AFC (level 2), defined as an AFC of ≥ 20 with a specificity of ≥ 95% (b). The same number of patients were diagnosed with PCOS according to the Rotterdam/IEBG 2023 criteria when applying serum AMH (level 2) and AFC (level 2) (c). [file JOG-52-0-s003.pptx]

## Slide 1
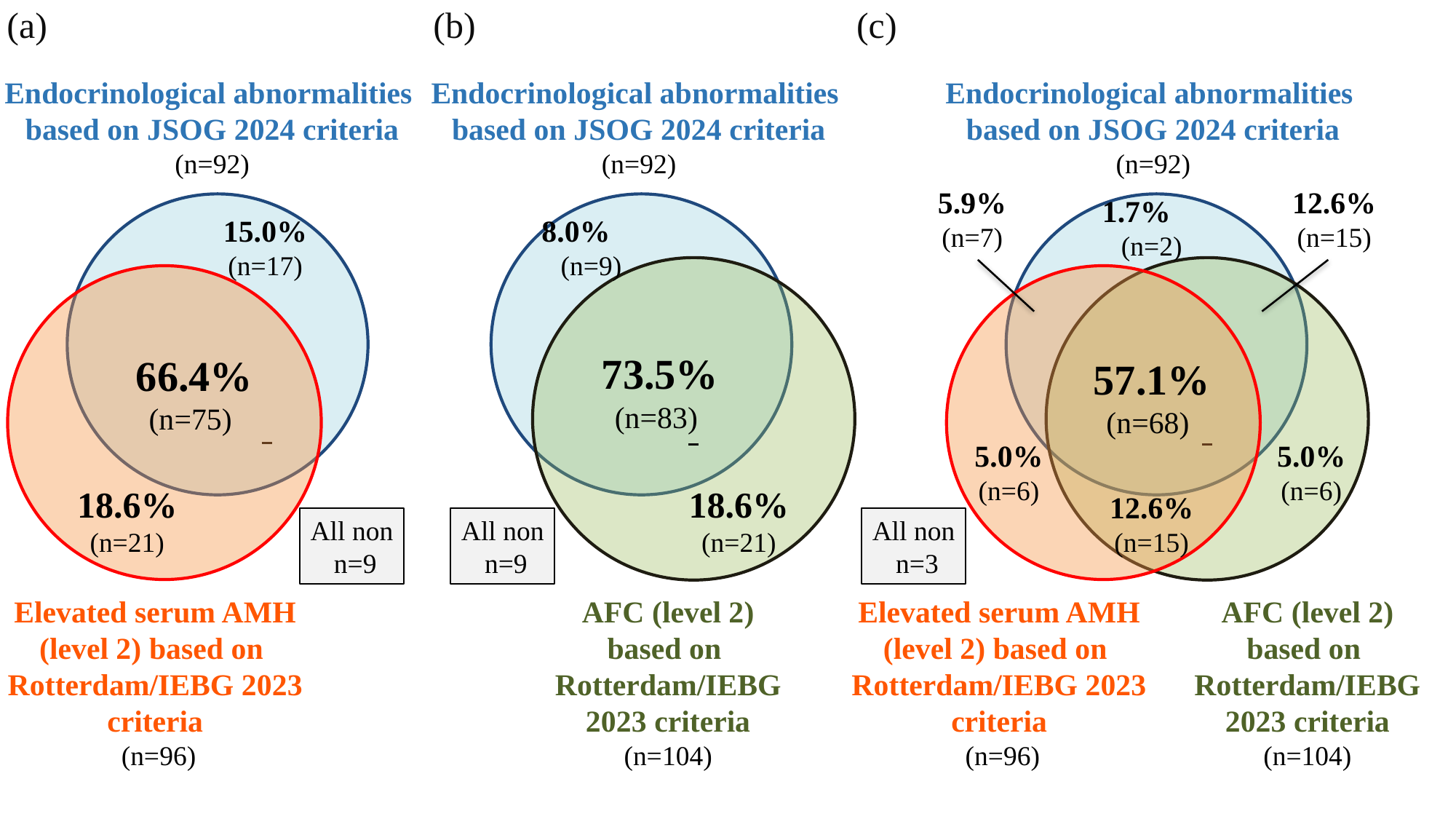

(a)
(b)
(c)
Endocrinological abnormalities
based on JSOG 2024 criteria
(n=92)
Endocrinological abnormalities
based on JSOG 2024 criteria
(n=92)
Endocrinological abnormalities
based on JSOG 2024 criteria
(n=92)
5.9%
(n=7)
12.6%
(n=15)
1.7%
(n=2)
15.0%
(n=17)
8.0%
(n=9)
73.5%
(n=83)
66.4%
(n=75)
57.1%
(n=68)
5.0%
(n=6)
5.0%
(n=6)
18.6%
(n=21)
18.6%
(n=21)
12.6%
(n=15)
All non
 n=9
All non
 n=9
All non
 n=3
Elevated serum AMH
(level 2) based on
Rotterdam/IEBG 2023 criteria
 (n=96)
AFC (level 2)
based on
Rotterdam/IEBG 2023 criteria
(n=104)
Elevated serum AMH
(level 2) based on
Rotterdam/IEBG 2023 criteria
 (n=96)
AFC (level 2)
based on
Rotterdam/IEBG 2023 criteria
(n=104)
